# Clinical diagnostic value of targeted next generation sequencing for lower respiratory tract infection: a retrospective study

**DOI:** 10.3389/fcimb.2026.1713445

**Published:** 2026-07-08

**Authors:** Yueyan Ni, Weiwei Wu, Jia Liu, Chunlai Feng, Bing Jin, Tingting Zhao, Yu Gu, Xin Su, Chong Li, Xin Yuan

**Affiliations:** 1 Department of Respiratory and Critical Care Medicine, Nanjing Drum Tower Hospital, Nanjing, China; 2 Department of Medicine, Dinfectome Inc., Nanjing, China; 3 Department of Respiratory and Critical Care Medicine, The First People’s Hospital of Changzhou, Changzhou, China; 4 Department of Respiratory and Critical Care Medicine, Changzhou Fourth People’s Hospital, Changzhou, China; 5 Department of Respiratory and Critical Care Medicine, Changzhou Third People’s Hospital, Changzhou, China

**Keywords:** conventional microbiological techniques, diagnosis, lower respiratory tract infections, structural lung diseases, targeted next-generation sequencing

## Abstract

**Objective:**

Lower respiratory tract infections (LRTIs) progress swiftly and require timely, accurate pathogen detection to enhance patient outcomes. This study aims to utilize the targeted metagenomic next-generation sequencing (tNGS) technology as a novel approach to investigate the types of pathogens involved in infections following different structural lung diseases.

**Methods:**

This retrospective cohort study enrolled 329 patients with suspected LRTIs admitted to three medical centers from June 2023 and June 2024. The study analyzed the pathogenic spectrum of lung infections and compared the diagnostic outcomes of tNGS with those of conventional microbiological techniques (CMTs).

**Results:**

tNGS demonstrated significantly higher sensitivity (97.8% vs. 28.9%, p<0.05) and accuracy (96.6% vs. 30.3%, p<0.05) than CMTs, along with a high concordance rate (87.8%) with clinically confirmed pathogens. Pathogen profiling revealed that *Mycoplasma pneumoniae* (21.88%), *Aspergillus fumigatus* (5.17%), and influenza A virus subtype H3N2 (13.07%) were the predominant bacterial, fungal, and viral pathogens, respectively. Several key pathogens, including *Pseudomonas aeruginosa*, *Haemophilus influenzae*, *Nocardia abscessus*, *Aspergillus fumigatus*, Influenza A virus H3N2, and Influenza B virus, were detected more frequently in the SLD group than in the non-SLD group. Among 193 patients whose treatment was adjusted based on tNGS results, 35.2% initiated new treatment regimens, 25.4% continued their original treatment, and 7.3% required treatment escalation, with 90.2% of these patients showing clinical improvement.

**Conclusion:**

These findings showed that tNGS demonstrates significant promise for the etiological diagnosis and tailored management of LRTIs.

## Introduction

Lower respiratory tract infections (LRTIs) remain a leading global health threat, disproportionately affecting children and older adults. In 2021, the research estimated 344 million incident episodes of LRTIs and 2.18 million deaths, among which 502 000 deaths were in children younger than 5 years (Bender et al., 2024). Conventional microbiological tests (CMTs) are limited by low sensitivity, lengthy turnaround times, and an inability to detect fastidious or non-culturable pathogens (Song et al., 2024; Elbehiry and Abalkhail, 2025). Consequently, empirical antibiotic therapy is common, driving antimicrobial resistance and prolonged hospitalization (Strich et al., 2020). The clinical implementation of metagenomic next-generation sequencing (mNGS) has markedly enhanced the pathogen diagnosis of LRTIs, particularly in cases of severe and refractory infections. This technological advancement has significantly optimized diagnostic efficiency, streamlined clinical decision-making, and improved patient prognoses (Chiu and Miller, 2019; Zou et al., 2025). However, its high sequencing depth, elevated human-host DNA burden, complex bioinformatics, and substantial cost have restricted routine clinical adoption (Li et al., 2021; Diao et al., 2022; Doualeh et al., 2022).

In this background, targeted next-generation sequencing (tNGS) has emerged as a cost-effective alternative to mNGS. It was specifically designed to retain the sensitivity and rapid turnaround time of mNGS while enhancing analytical specificity and reducing sequencing costs (Sun et al., 2024). By utilizing multiplex PCR or hybrid-capture probes to enrich nucleic acids from hundreds of clinically relevant pathogens and resistance genes, tNGS not only exhibits a higher detection sensitivity but also achieves a shorter turnaround time and reduced costs compared to mNGS (Fu and Zhou, 2024; Yin et al., 2024). Nevertheless, robust clinical-performance data for tNGS in LRTIs remain scarce. The present study aims to evaluate the clinical utility of tNGS in diagnosing LRIs and characterize the local pathogen spectrum, by comparing its performance with CMTs such as culture, pharyngeal swab PCR, and antigen-antibody assays.

## Methods

### Study design and participants

**Figure 1:**
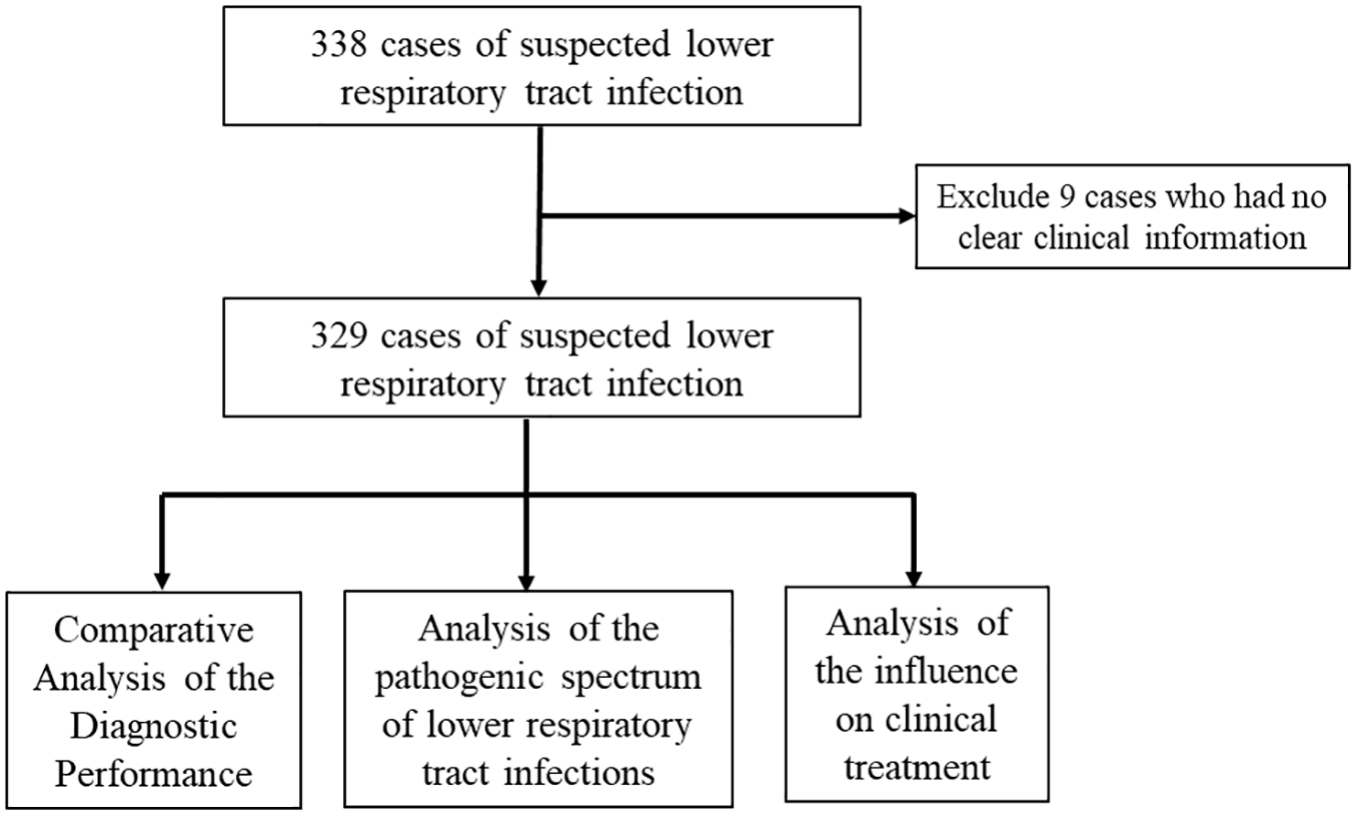
Overview of patient enrollment workflow.

This retrospective study included patients with suspected LRTIs who were hospitalized in Nanjing Drum Tower Hospital, Changzhou First People’s Hospital and Changzhou Fourth People’s Hospital from June 2023 to June 2024. The inclusion criteria were: (1) age ≥ 18 years; (2) clinical suspicion of LRTIs; and (3) collection of sputum, bronchoalveolar lavage fluid (BALF), or pleural fluid within 48 hours of admission for both tNGS and CMTs. Patients with incomplete clinical records or insufficient sample volume for both tests were excluded. This study was conducted with the approval of the Nanjing Drum Tower Hospital Ethics Committee (No.202347802) and with the informed consent of all participants and their legal guardians. All procedures were carried out in compliance with the relevant guidelines and regulations. The study flow is depicted in **Figure 1**.

### Clinical microbiologic diagnostics

The final attribution of causative pathogens was determined by the attending physician in consultation with at least two senior microbiology or infectious disease specialists, based on clinical manifestations, radiological findings, inflammatory biomarkers, microbiological results from both tNGS and CMTs, biological plausibility, sample quality, host immune status, underlying pulmonary diseases, and response to pathogen-directed therapy. Organisms more likely to represent colonization, contamination, or commensal flora, as well as microbes reported by the clinical laboratory as laboratory, skin, or environmental contaminants or as mixed upper respiratory flora, were excluded when inconsistent with the overall clinical context (Mick et al., 2023; Zou et al., 2025). Disagreements were resolved through consensus discussion.

### Sample collection and clinical data acquisition

A total of 329 clinical specimens were collected for pathogen testing, including 320 samples of BALF and 9 sputum samples. For BALF collection, topical pharyngeal anesthesia was administered before fiberoptic bronchoscopy. The bronchoscope was advanced to the target lesion area, and bronchoalveolar lavage was performed according to standard procedures. The recovered lavage fluid was aspirated and collected into sterile containers. For sputum collection, patients were instructed to perform oral hygiene and then cough deeply from the lungs to produce sputum, which was collected in a sterile container. If expectoration was difficult, inhalation of sterile saline via a nebulizer was used to induce sputum production. After collection, specimens were immediately divided for parallel testing. CMTs were performed in the hospital laboratory according to routine clinical practice, while aliquots for tNGS were sent to Dinfectome Inc. for sequencing analysis. Demographic characteristics, clinical features, and laboratory findings were extracted from the electronic medical records through the hospital information system. Data on antimicrobial exposure before admission, initial antimicrobial regimens after admission, and subsequent treatment modifications potentially guided by tNGS results were also collected.

### Conventional microbiological tests

The collected samples were immediately subjected to a series of laboratory tests immediately in the hospital laboratory according to routine clinical practice. Conventional diagnostic methods included blood and sputum cultures, smear microscopy, and bacterial culture. Serological and antigen-based assays were also performed when clinically indicated, including the serum (1,3)-β-D-glucan test, galactomannan test, and Mycoplasma pneumoniae antibody detection. For suspected tuberculosis, acid-fast staining and Xpert MTB/RIF assay were used for the detection of *Mycobacterium tuberculosis*. In addition, quantitative polymerase chain reaction (qPCR) was performed to detect respiratory viral nucleic acids and other clinically suspected pathogens.

### tNGS test

#### Nucleic acid extraction, library preparation and sequencing

After the clinical samples were pretreated, nucleic acid extraction was performed using the MagMAX Pathogen RNA/DNA Kit (Thermo Fisher Scientific) following the manufacturer’s protocols. The quantity and quality of nucleic acids were assessed using the Qubit (Thermo Fisher Scientific) and NanoDrop (Thermo Fisher Scientific), respectively. Following cDNA synthesis and multiplex PCR preamplification of specific target loci, library preparation was conducted using the Hieff NGS C130P2 OnePot II DNA Library Prep Kit for MGI (Yeasen Biotechnology) in accordance with the manufacturer’s protocols. Agilent 2100 was used for quality control and libraries were 50bp single-end sequenced on DIFSEQ-200.

#### Bioinformatics analysis

Raw sequencing data were splited by bcl2fastq2 (version 2.20), and high-quality sequencing data were generated using Trimmomatic (version 0.36) by removing low quality reads, non-specific primer binding, adapter contamination, duplicated and shot (length<36 bp) reads. Human host sequences were subtracted by mapping to human reference genome (hs37d5) using bowtie2 (version 2.2.6). Reads that could not be mapped to the human genome were retained and aligned with microorganism genome database using bowtie2 (version 2.2.6). The reads with 95% identity of reference were defined as mapped reads. DIAMOND was used mapping Comprehensive Antibiotic Resistance Database (CARD) to identify antimicrobial resistance genes (ARGs). ARG-associated reads were taxonomically classified against a microbial reference database to infer the source organism.

### Interpretation and reporting

We used the following criteria for positive results of tNGS: (1) For *Mycobacterium*, the result was considered positive if the species detected by tNGS had a species-specific read number≥200; (2) For Human betaherpesvirus 6, Human betaherpesvirus 7 and Human gammaherpesvirus 4, the result was considered positive if the species detected by tNGS had a species-specific read number≥1000; (3) For bacteria (excluding Mycobacterium), fungi, virus (excluding Human betaherpesvirus 6, Human betaherpesvirus 7 and Human gammaherpesvirus 4) and parasites, the result was considered positive if a species detected by tNGS had a species-specific read number≥30.

### Statistical analysis

Continuous variables are presented as mean ± standard deviation and compared using Student’s t-tests for normally distributed data, while non-normally distributed data are reported as median (interquartile range) and analyzed using the Mann-Whitney U test. Statistical analyses were conducted using R version 4.1.2 (R Foundation for Statistical Computing, Vienna, Austria). A P-value < 0.05 was considered statistically significant.

### Diagnostic performance assessment

Using the adjudicated composite clinical reference standard rather than a single microbiological gold standard, we evaluated the diagnostic performance of tNGS and CMTs. A true-positive result was defined as the detection of at least one clinically adjudicated causative pathogen. A false-positive result was defined as the detection of an organism considered to represent colonization, contamination, or a clinically irrelevant finding, or any positive result in patients ultimately adjudicated as having a non-infectious condition. A true-negative result was defined as the absence of pathogen detection in patients adjudicated as having no infectious etiology. A false-negative result was defined as failure of the assay to detect a clinically adjudicated causative pathogen.

### Treatment response assessment

Treatment responses were classified as clinical improvement, deterioration, unchanged, death, or untraceable according to the Chinese guidelines for the diagnosis and treatment of adult community-acquired pneumonia and medical record review. Clinical improvement was defined as resolution or substantial alleviation of infection, indicated by decreased inflammatory markers, radiological improvement on chest CT, and relief of respiratory symptoms. Deterioration was defined as clinical or radiological worsening of infection. Unchanged status was defined as persistent symptoms without meaningful improvement in inflammatory markers or imaging findings, but without clear progression. Death was defined as in-hospital mortality during the index admission. Untraceable were considered when treatment response could not be reliably assessed because of transfer, discharge against medical advice, poor adherence, or incomplete medical records.

## Results

### Clinical characteristics of patients

A total of 329 patients with suspected lower respiratory tract infection were finally enrolled in this study, including 182 males (55.3%) and 147 females (44.7%), and the median age was 61 (IQR, 48-71) years. The demographic characteristics of the included patients are shown in the **Table 1**. 196 (59.57%) patients had underlying diseases before admission. Among them, 102 patients had underlying lung diseases, including bronchiectasis (13.07%), chronic obstructive pulmonary disease (COPD, 6.08%), interstitial pneumonia (3.95%), and other lung diseases (7.29%). In addition, the more common comorbidities were diabetes (29.5%) and cancer (10.94%).

**Table 1:** Clinical characteristic of samples in present study (n = 329).

Characteristic	N=329
Sex (male)	182 (55.3%)
Age, y, median (IQR)	61(48,71)
<18	14 (4.26%)
18-44	56 (17.02%)
45-65	120 (36.47%)
>65	127 (38.60%)
Unknown	12 (3.65%)
Underlying disease	196 (59.57%)
Bronchiectasis	43 (13.07%)
Cancer	36 (10.94%)
Diabetes	26 (7.90%)
Other lung diseases	26 (7.90%)
Chronic obstructive pulmonary disease	20 (6.08%)
Hypertension	14 (4.26%)
Interstitial pneumonia	13 (3.95%)
Other chronic diseases	12 (3.65%)
Rheumatic disease	9 (2.74%)
Coronary heart disease	7 (2.13%)

### The diagnostic performance comparison between tNGS and CMT

**Figure 2:**
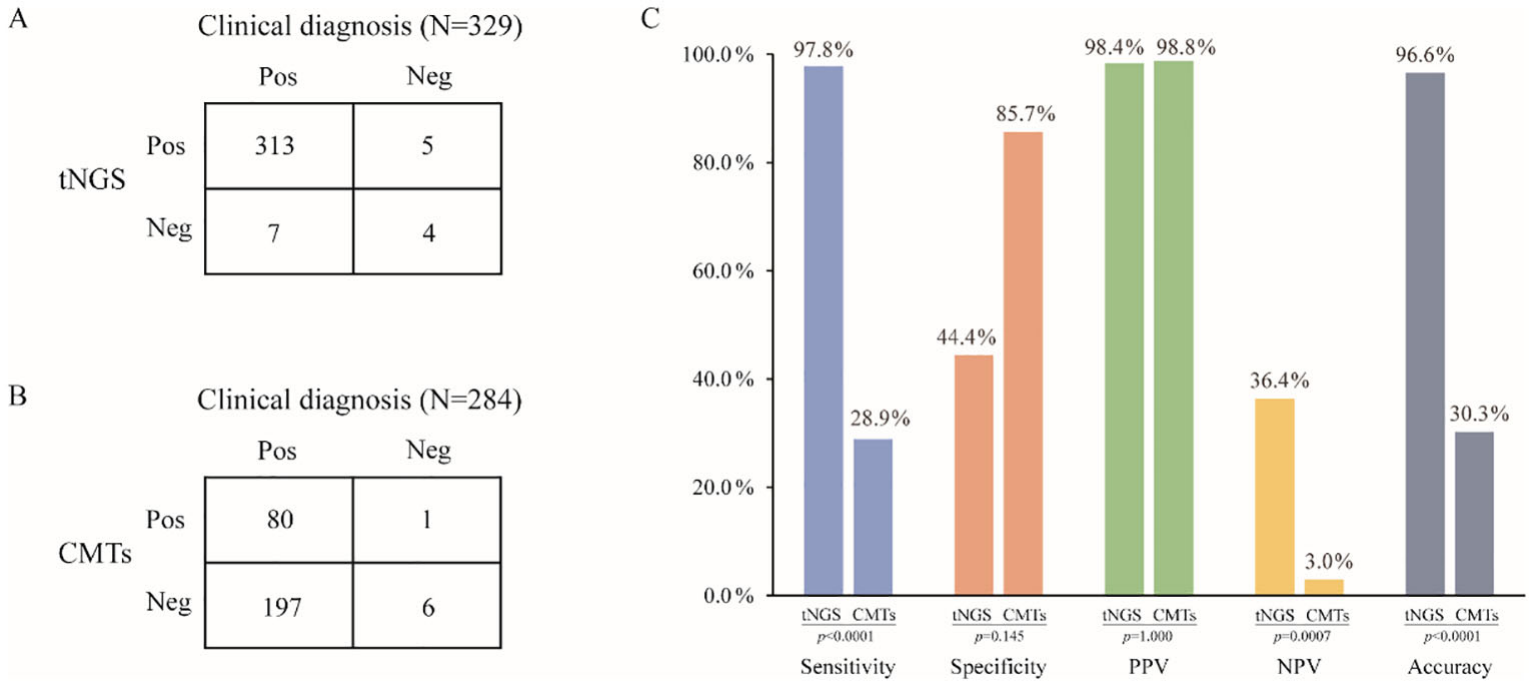
The diagnostic performance comparison between tNGS and CMTs. **(A)** Diagnostic performance of tNGS compared with the clinical diagnosis. **(B)** Diagnostic performance of CMTs compared with the clinical diagnosis. **(C)** Comparison of sensitivity, specificity, positive predictive value, negative predictive value, and accuracy between tNGS and CMTs.

In this study, using clinical diagnosis as the composite clinical reference standard, the diagnostic performance of tNGS and CMT was compared (**Figure 2**). tNGS demonstrated a superior sensitivity of 97.8% [95% CI (95.6-99.0%)] for diagnosing lower respiratory tract infections, significantly higher than CMTs’ 28.9% [95% CI (20.6-38.2%); p<0.05], with a statistically significant difference (p<0.05). However, tNGS showed lower specificity [44.4% (95% CI 24.2–64.6%)] compared to CMTs’ 85.7% [95% CI (62.5–95.5%); p<0.05]. The positive predictive value (PPV) of tNGS was 98.4% [95% CI (96.0–99.3%)], slightly lower than that of CMT at 98.8% [95% CI (91.6–99.7%)]. In contrast, the negative predictive value (NPV) of tNGS was 36.4% [95% CI (16.1–57.9%)], significantly higher than CMT’s 3.0% [95% CI (0.4–16.1%)]. The overall diagnostic accuracy of tNGS was 96.6% [95% CI (93.9–98.3%)], markedly higher than CMT’s 30.3% [95% CI (20.6–40.0%)].

### Pathogen spectrum of patients with lower respiratory tract infections

**Figure 3:**
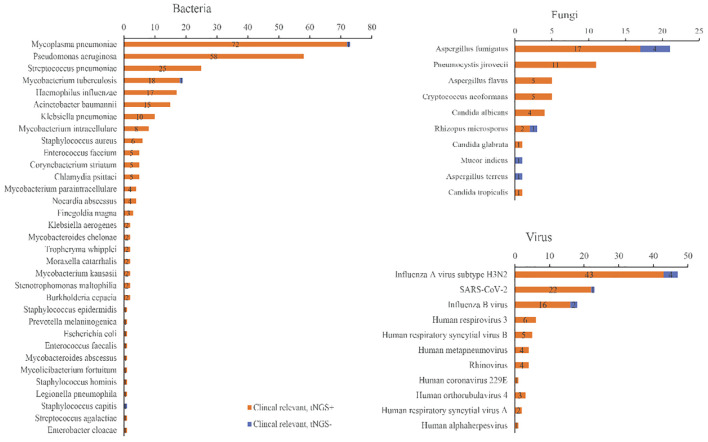
Comparison of tNGS and CMTs on pathogenic microorganisms.

**Figure 3** illustrated the definitive clinical pathogen spectrum in this study. Among patients with LRTIs, the predominant bacterial pathogens were *Mycoplasma pneumoniae* (21.88%), *Pseudomonas aeruginosa* (17.63%), *Streptococcus pneumoniae* (7.60%), *Mycobacterium tuberculosis* (5.47%), and *Haemophilus influenzae* (5.17%). The common fungal pathogens included *Aspergillus fumigatus* (5.17%), *Pneumocystis jirovecii* (3.34%), *Aspergillus flavus* (1.52%), and *Cryptococcus neoformans* (1.52%). The viral agents were mainly dominated by Influenza A virus subtype H3N2 (13.07%), SARS-CoV-2 (6.69%), and Influenza B virus (4.86%). This pathogen profile demonstrates the substantial diagnostic utility of tNGS in LRTI pathogen identification, particularly for atypical pathogens such as Nontuberculous Mycobacteria (NTM), Mucorales, viral agents, Nocardia, and *Chlamydia psittaci*, where tNGS exhibited irreplaceable diagnostic value. However, tNGS also exhibited missed detections of pathogens in certain samples, such as *Aspergillus fumigatus* (4/21), Influenza A virus subtype H3N2 (4/47), and Influenza B virus (2/18).

### Evaluation of tNGS and CMT concordance with clinical diagnoses

**Figure 4:**
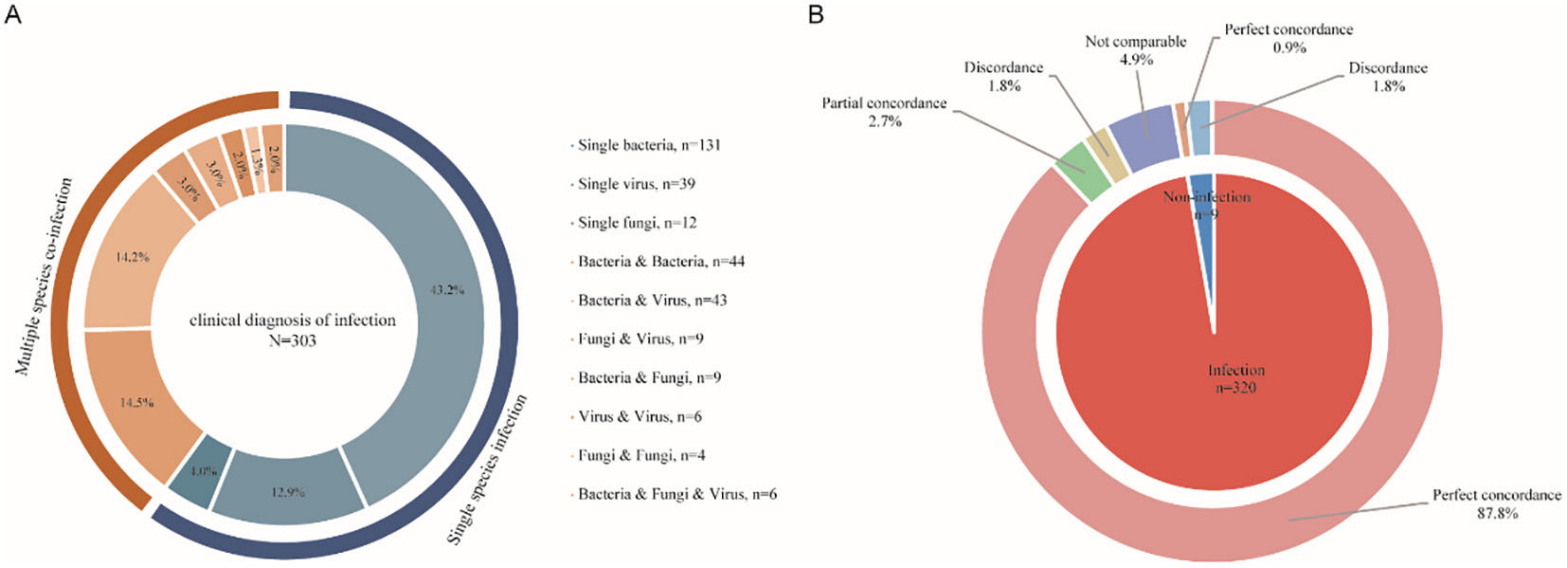
Analysis of infection types and diagnostic concordance. **(A)** Pie diagram of the distribution of detected pathogens. **(B)** Comparison of the consistency between tNGS and clinically definitive pathogens.

Among the 303 clinically diagnosed patients with definite infectious pathogens, single-species infections accounted for 60.1%, while mixed infections with two or more species made up 39.9%. Within the mixed infection subgroup, infections of a single pathogen category represented 17.8%, and co-infections involving bacteria, fungi, and viruses were observed in 2.0% of cases (**Figure 4A**).

The concordance between tNGS and CMTs results relative to clinical diagnoses was evaluated (**Figure 4B**; **Supplementary Figure 1**). Among the 320 patients with clinically confirmed infections, comparison between tNGS detection results and clinically definitive pathogens revealed complete concordance in 87.8% of cases, partial concordance in 2.7%, discordance in 1.8%. Notably, 4.9% of samples were non-comparable due to absence of clinically confirmed pathogenic microorganisms in some patients. In 9 patients with definitive non-infectious diagnoses, only 0.9% (3 patients) exhibited complete concordance, whereas 1.8% exhibited discordant results. Moreover, Among the 81 samples that tested positive by CMTs, complete concordance between tNGS and CMT results was observed in 72.84% of cases.

### Comparison of pathogens in patients with structural lung diseases

**Figure 5:**
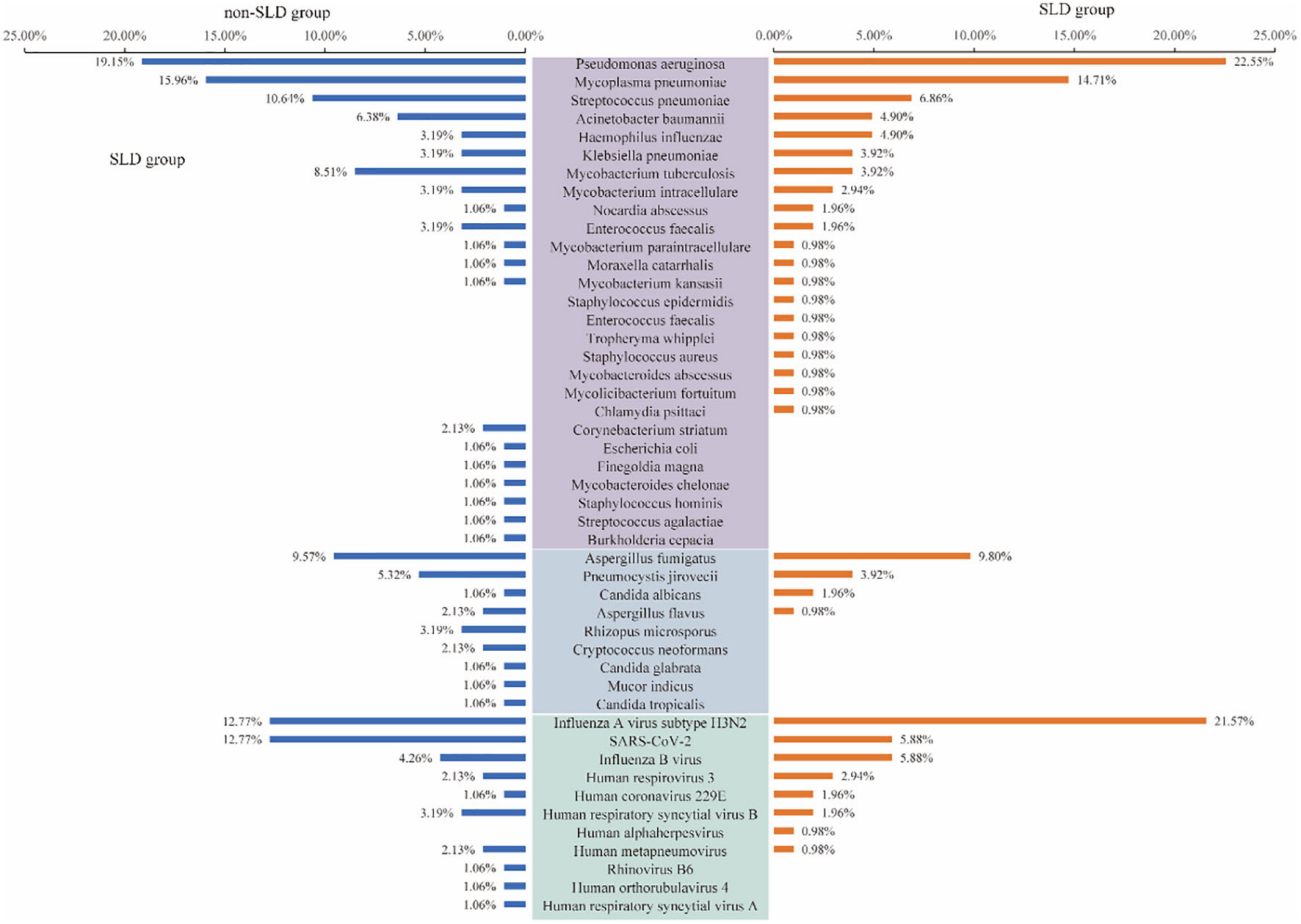
Pathogen distribution detected by tNGS in patients with and without structural lung diseases. SLD, structural lung diseases.

Patients with confirmed LRTIs were categorized into structural lung disease (SLD) cohort (n=102) and non-structural lung diseases (non-SLD) cohort (n=94) based on structural pulmonary abnormalities (**Figure 5**). Comparative analysis between the two cohorts revealed distinct pathogen distribution patterns. Notably, the detection rates of several key pathogens, such as *Pseudomonas aeruginosa*, *Haemophilus influenzae*, *Nocardia abscessus*, *Aspergillus fumigatus*, Influenza A virus H3N2, and Influenza B virus, were elevated in the SLD group relative to the non-SLD group. In addition, *Chlamydia psittaci*, *Enterococcus faecalis*, and *Mycobacterium abscessus* were exclusively detected in the SLD group. In contrast, *Corynebacterium striatum*, *Rhizopus microsporus*, *Cryptococcus neoformans*, and *Mucor indicus* were only identified in the non-SLD group. Among viruses, Rhinovirus B6, Human orthorubulavirus 4, and Human respiratory syncytial virus A were also solely found in the non-SLD cohort.

### The pathogen profiles of different subgroups

**Figure 6:**
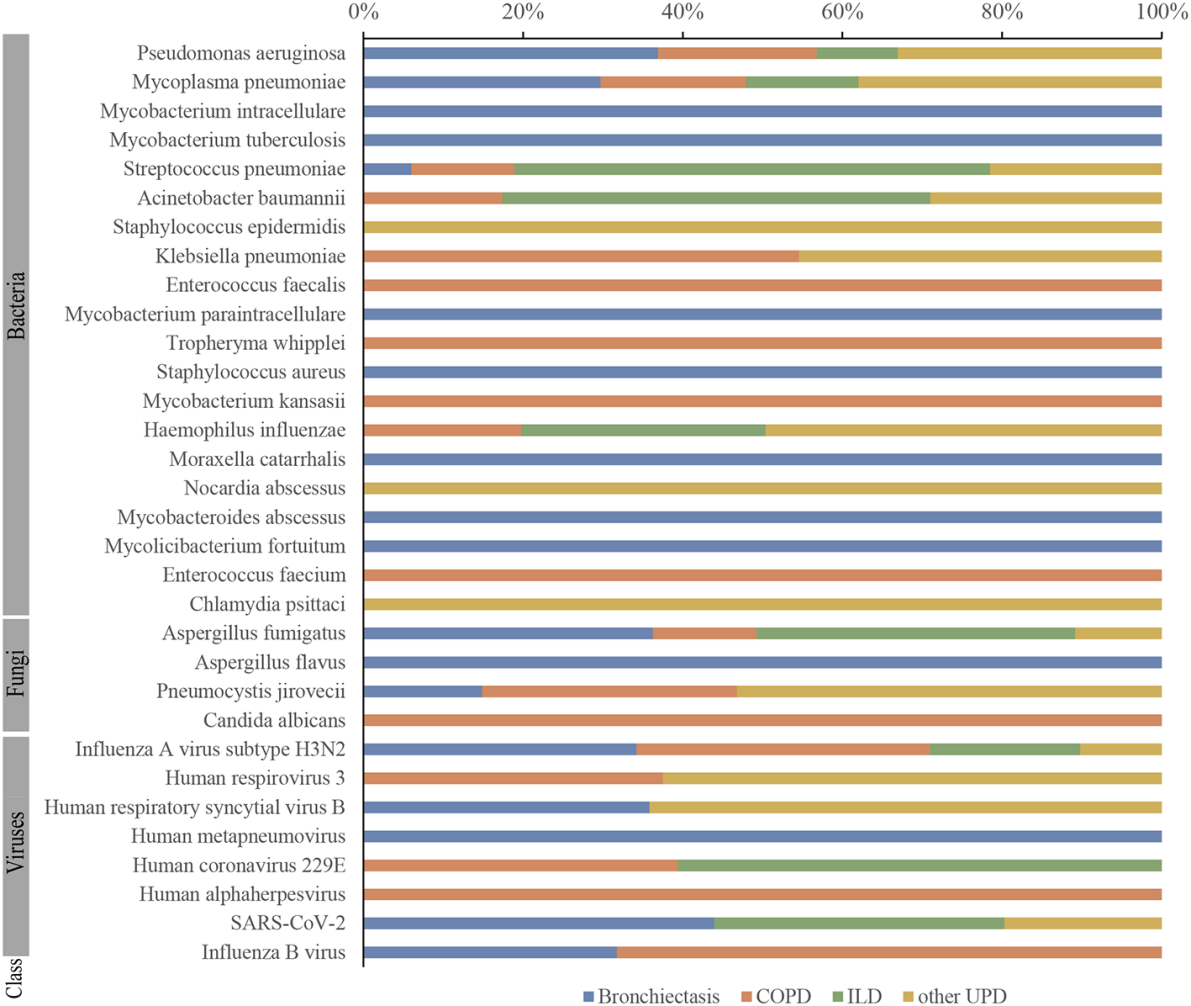
Pathogen distribution detected by tNGS across different structural lung disease subgroups. COPD, chronic obstructive pulmonary disease; ILD, interstitial pneumonia; UPD, underlying pulmonary disease.

We conducted a statistical analysis of pathogenic microorganism detection across various subgroups, including those with COPD (n=20), interstitial pneumonia (n=13), bronchiectasis (n=43), and other lung diseases (n=26) (**Figure 6**). Patients with bronchiectasis exhibited a broader spectrum of pathogens, while those with interstitial pneumonia showed a more evenly distributed pathogen profile. *Mycoplasma pneumoniae*, *Streptococcus pneumoniae*, *Pseudomonas aeruginosa*, *Aspergillus fumigatus*, and Influenza A virus were detected across all subgroups. Among them, the detection rate of *Pseudomonas aeruginosa* in patients with bronchiectasis and those with COPD was 27.91% and 15% respectively. Notably, *Mycobacterium tuberculosis* had a detection rate of 6.98% in patients with bronchiectasis.

### Value of tNGS results for clinical management

**Figure 7:**
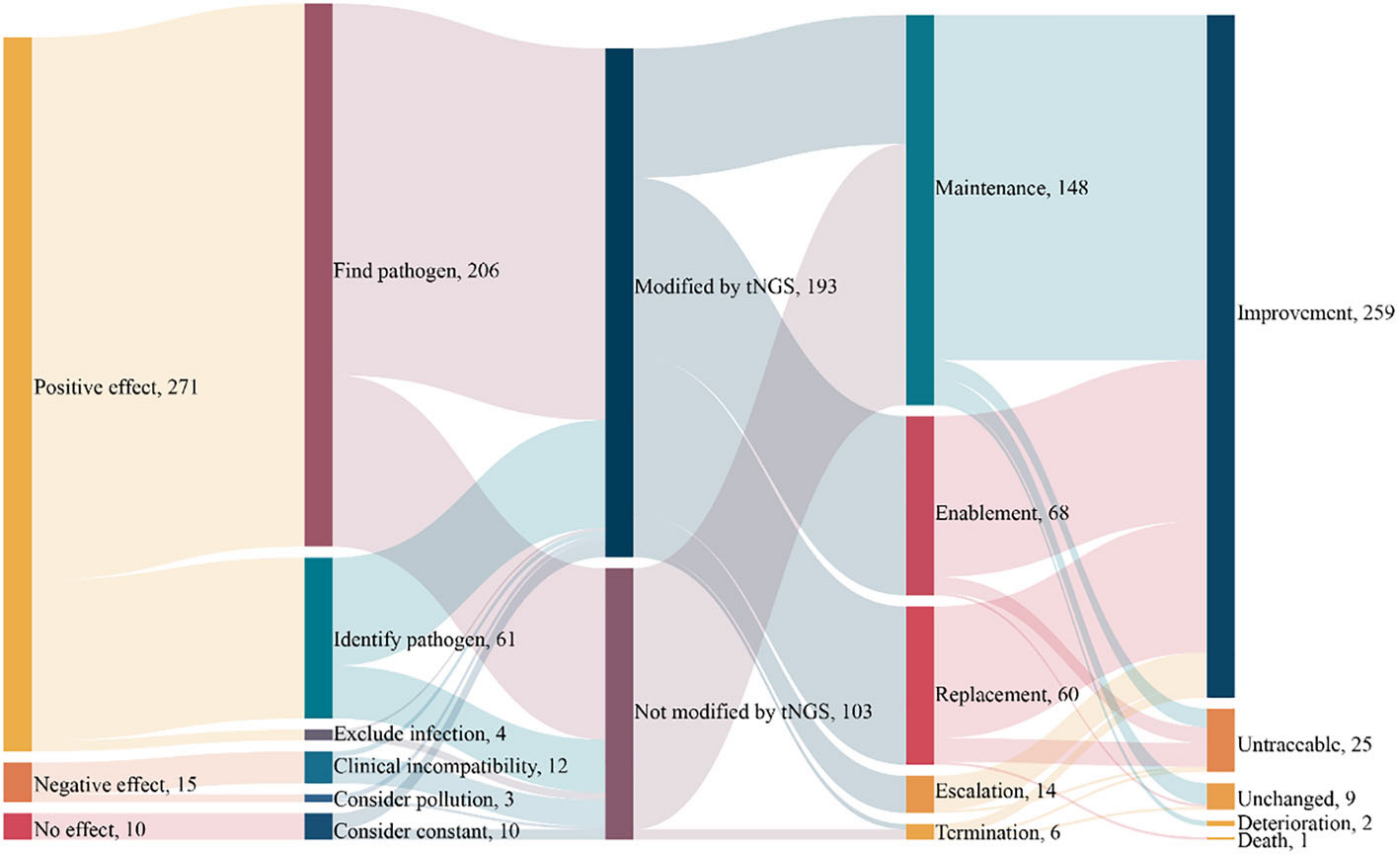
tNGS-directed treatment modalities in patients.

The impact of tNGS results on subsequent clinical management and outcomes was evaluated in this study (**Figure 7**). Among the patients, tNGS results had a positive influence on treatment decisions in 271 cases, a negative impact in 15 cases, and no impact in 10 cases, respectively. In terms of diagnostic significance, tNGS facilitated the detection of pathogen in 206 patients, identification of the pathogen in 61 patients, and exclusion of infection in four patients. However, the results were inconsistent with clinical manifestations in 12 cases. Of the 193 patients whose treatment plans were adjusted based on tNGS results, 68 (35.2%) initiated a new treatment regimen, 49 (25.4%) continued the original treatment, and 14 (7.3%) escalated treatment. Among those who had their treatment adjusted based on tNGS, 90.2% showed clinical improvement, 8.3% worsened, two had no change, and one patient died. These findings underscore the potential of tNGS to guide clinical decision-making and improve patient outcomes, particularly in cases where traditional methods fail to provide timely or accurate diagnostic information.

### Analysis results of drug-resistant genes

In our study, 7 drug-resistant genes were reported in the 44 samples by tNGS (**Table 2**; **Supplementary Table 1**). The most frequently detected resistance gene was OXA-51, which was identified in 17 samples, all of which were from *Acinetobacter baumannii*. The Mycoplasma pneumoniae 23S rRNA mutation A2063G and the OXA-23 gene were the next most common, being detected in 15 and 14 samples, respectively. It was also found that the same resistance genes can be present in multiple species. For example, the mecA gene was mainly from *Staphylococcus aureus*, the KPC gene was from *Pseudomonas aeruginosa* and *Klebsiella pneumoniae*, and the NDM gene was from *Acinetobacter baumannii*, *Enterobacter cloacae*, *Klebsiella pneumoniae*, and *Proteus mirabilis*. Except for the Mycoplasma pneumoniae 23S rRNA mutation A2063G and OXA-23, the resistance mechanisms of the other detected genes were mainly antibiotic inactivation.

**Table 2:** Drug resistant gene and pathogens detected by tNGS.

Drug resistance gene	Detected number	Species	Mechanism
*OXA-51*	17	*Acinetobacter baumannii*	Antibiotic inactivation
*Mycoplasma pneumoniae 23S rRNA mutation A2063G*	15	*Mycoplasma pneumoniae*	
*OXA-23*	14	*Acinetobacter baumannii*	Antibiotic target replacement
*mecA*	12	*Staphylococcus aureus, Staphylococcus haemolyticus*	Antibiotic inactivation
*KPC*	5	*Pseudomonas aeruginosa, Acinetobacter baumannii, Klebsiella aerogenes, Escherichia coli, Klebsiella oxytoca, Klebsiella pneumoniae, Morganella morganii, Proteus mirabilis*	Antibiotic inactivation
*NDM*	5	*Pseudomonas aeruginosa, Acinetobacter baumannii, Klebsiella aerogenes, Enterobacter cloacae, Escherichia coli, Klebsiella pneumoniae, Morganella morganii, Proteus mirabilis*	Antibiotic inactivation
*CTX-M*	4	*Klebsiella aerogenes, Escherichia coli, Klebsiella pneumoniae, Proteus mirabilis, Salmonella enterica*	Antibiotic inactivation

## Discussion

Lower respiratory tract infections are characterized by rapid onset and progression, and early, precise pathogen identification is critical for improving patient outcomes. Traditional detection methods often result in diagnostic delays due to their lengthy testing cycles and low sensitivity. While mNGS is widely used in clinical practice, its application in lower respiratory tract infections is still challenging due to high costs and human sequence interference (Diao et al., 2022). In contrast, as a molecular detection method based on targeted amplification and high-throughput sequencing, tNGS can overcome some of the limitations of mNGS in pathogen diagnosis. tNGS is not affected by the human genome and background microbiota, and it offers several advantages, including lower costs, minimal sample requirements, easy workflow standardization, and the ability to detect both DNA and RNA pathogens simultaneously (Ding et al., 2025).

In this study, tNGS demonstrated significantly higher sensitivity (97.8%) and accuracy (96.6%) than CMTs when using clinical diagnosis as the reference standard, which is consistent with previous studies highlighting the high sensitivity of tNGS in infectious disease diagnosis (Ma et al., 2024; Yang et al., 2025). However, the specificity of tNGS was relatively low (44.4%), possibly due to false-positive or clinically irrelevant detections, including colonizing organisms, contaminants, or nucleic acids from nonviable pathogens. These findings indicated that tNGS results should not be used as a standalone basis for treatment decisions, but should be interpreted in combination with clinical manifestations, radiological findings, inflammatory markers, and CMT results (Yang et al., 2025). In clinical practice, tNGS may be particularly valuable for patients with severe or refractory LRTIs, prior antimicrobial exposure, negative conventional tests despite high clinical suspicion, suspected atypical or fastidious pathogens, or possible mixed infections.

In pathogen spectrum construction, tNGS detected various common pathogens, such as *Mycobacterium tuberculosis*, *Legionella pneumophila*, and *Cryptococcus neoformans*, showing high consistency with clinical diagnosis and confirming its ability to identify highly pathogenic pathogens. However, tNGS failed to detect certain pathogens like *Mucor indicus*, *Aspergillus fumigatus*, and Influenza A virus H3N2. The possible reasons include: first, the thick cell walls of fungi may reduce detection efficiency due to insufficient cell wall disruption during nucleic acid extraction (Karakousis et al., 2006; Yuan et al., 2025); second, genetic variation in some viruses (e.g., Influenza A virus) may affect probe binding efficiency (Emery et al., 2000); and third, inappropriate sample collection timing (e.g., low pathogen loads early in the disease course) (Rajapaksha et al., 2019). In the future, we need to optimize the nucleic acid extraction method and update the coverage of the probe library to reduce the leakage and improve the accuracy of the test.

Subgroup analyses revealed significant differences in pathogen profiles between patients with structural lung diseases (e.g., COPD, bronchiectasis) and those with nonstructural diseases. For instance, *Chlamydia psittaci* and *Mycobacterium abscessus* were exclusively detected in the structural lesion group, while *Corynebacterium striatum* and *Cryptococcus neoformans* were only found in the nonstructural lesion group. These differences may stem from the distinct pathophysiological characteristics and immune statuses of the patient groups (Beasley et al., 2012). Furthermore, the detection rate of *Pseudomonas aeruginosa* in the SLD group was significantly higher than that in the non-SLD group, which aligns with prior research indicating that *Pseudomonas aeruginosa* is frequently detected among patients with structural respiratory diseases (Yang et al., 2023). This suggested that tNGS can refine pathogen detection by considering patients’ underlying conditions, offering personalized diagnostic guidance for different subgroups. Notably, *Enterococcus faecalis* was detected only in the COPD group. Although it can cause nosocomial infections in immunocompromised patients (Sangiorgio et al., 2024), its detection in respiratory specimens should be interpreted cautiously and may indicate colonization or contamination in the absence of supportive clinical evidence. Additionally, differences in pathogen profiles among patients with distinct comorbidities, such as interstitial pneumonia, may provide valuable evidence for developing targeted prevention and treatment strategies.

In clinical practice, tNGS results played a positive role in 91.56% of patients, with tNGS enabling pathogen detection in 76% of patients, which is consistent with previous research findings (Li et al., 2025). Moreover, tNGS guided treatment modifications in 193 patients, including the initiation of new treatment regimens, maintenance of existing treatments, or intensification of therapy. This showed tNGS helps to realize the shift from empirical treatment to precision medicine. In the management of lower respiratory tract infections, it provides clinicians with pathogen and drug resistance-related information. This prevents under-treatment due to underdiagnosis and over-treatment due to misdiagnosis, and is particularly useful in complex situations such as multi-pathogen or severe infections (Zhang et al., 2024).

The dominant resistance determinants identified in this study were OXA-51 (in *Acinetobacter baumannii*) and 23S rRNA mutations (in *Mycoplasma pneumoniae*), both of which confer resistance via antibiotic inactivation or target-site alteration, consistent with well-established mechanisms (Lopes et al., 2012; Vester and Long, 2013). These findings demonstrate that tNGS coupled with resistance-gene profiling enables simultaneous “pathogen–resistance” characterization, offering a streamlined, one-stop solution for precision antimicrobial therapy—particularly critical for infections caused by multidrug-resistant organisms. The results underscore the imperative of integrating resistance surveillance into routine clinical management and provide a foundation for future precision-driven antimicrobial strategies.

Although our study provided valuable insights into the application of tNGS in lower respiratory tract infections, several limitations should be acknowledged. First, despite participants were recruited from three centers, most were enrolled within a single geographic region, which may introduce selection bias. Multicenter, prospective cohorts spanning diverse climatic zones and healthcare settings are warranted to validate the generalizability of our findings. Second, the interstitial pneumonia subgroup was under-represented, resulting in wide confidence intervals and limited statistical power to detect meaningful differences. In addition, AMR genes detected by tNGS were not systematically validated by phenotypic antimicrobial susceptibility testing or MIC determination. Future prospective studies with pre-specified sample size calculations for each subgroup, and concurrent collection of antimicrobial susceptibility data, are required to better delineate the pathogen spectrum and its clinical implications across distinct patient populations.

In summary, tNGS demonstrates considerable promise for the etiological diagnosis and tailored management of lower respiratory tract infections. Although there are still some limitations in terms of specificity and detection range, the performance and clinical value of tNGS are expected to be further improved through the continuous optimization of the technical process and clinical application specifications. In the future, we look forward to larger, multicenter studies to further validate and expand the use of tNGS in lower respiratory tract infections, and to promote its widespread use in clinical practice to bring better treatment outcomes and prognosis to patients.

## Data Availability

All sequence reads were deposited into the Genome Sequence Archive in the National Genomics Data Center under the accession number PRJCA067218.
