# Supplementary material for: Clinical diagnostic value of targeted next generation sequencing for lower respiratory tract infection: a retrospective study
Source: Front Cell Infect Microbiol. 2026 Jul 8;16:1713445. doi: 10.3389/fcimb.2026.1713445 (PMC13388218; doi:10.3389/fcimb.2026.1713445)
Supplement: Supplementary file 1 [file Table1.docx]

Supplementary Table1 The resistance genes and related species detected in samples

| Sample.No | Drug resistance gene | Detected species |
| --- | --- | --- |
| N034 | CTX-M | Proteus mirabilis |
| N047 | CTX-M | Escherichia coli |
| N075 | CTX-M | Klebsiella pneumoniae |
| N178 | CTX-M | Escherichia coli |
| N035 | KPC | Pseudomonas aeruginosa, Klebsiella pneumoniae |
| N074 | KPC | Pseudomonas aeruginosa |
| N190 | KPC | Pseudomonas aeruginosa |
| N218 | KPC | Pseudomonas aeruginosa, Klebsiella pneumoniae |
| N031 | mecA | Staphylococcus aureus |
| N032 | mecA | Staphylococcus aureus |
| N035 | mecA | Staphylococcus aureus |
| N172 | mecA | Staphylococcus aureus |
| N178 | mecA | Staphylococcus aureus |
| N183 | mecA | Staphylococcus aureus |
| N191 | mecA | Staphylococcus aureus |
| N194 | mecA | Staphylococcus aureus |
| N200 | mecA | Staphylococcus aureus |
| N207 | mecA | Staphylococcus aureus |
| N313 | mecA | Staphylococcus aureus |
| N327 | mecA | Staphylococcus aureus |
| N046 | Mycoplasma pneumoniae 23S rRNA mutation:A2063G | Mycoplasma pneumoniae |
| N048 | Mycoplasma pneumoniae 23S rRNA mutation:A2063G | Mycoplasma pneumoniae |
| N087 | Mycoplasma pneumoniae 23S rRNA mutation:A2063G | Mycoplasma pneumoniae |
| N166 | Mycoplasma pneumoniae 23S rRNA mutation:A2063G | Mycoplasma pneumoniae |
| N169 | Mycoplasma pneumoniae 23S rRNA mutation:A2063G | Mycoplasma pneumoniae |
| N173 | Mycoplasma pneumoniae 23S rRNA mutation:A2063G | Mycoplasma pneumoniae |
| N183 | Mycoplasma pneumoniae 23S rRNA mutation:A2063G | Mycoplasma pneumoniae |
| N188 | Mycoplasma pneumoniae 23S rRNA mutation:A2063G | Mycoplasma pneumoniae |
| N191 | Mycoplasma pneumoniae 23S rRNA mutation:A2063G | Mycoplasma pneumoniae |
| N302 | Mycoplasma pneumoniae 23S rRNA mutation:A2063G | Mycoplasma pneumoniae |
| N306 | Mycoplasma pneumoniae 23S rRNA mutation:A2063G | Mycoplasma pneumoniae |
| N313 | Mycoplasma pneumoniae 23S rRNA mutation:A2063G | Mycoplasma pneumoniae |
| N315 | Mycoplasma pneumoniae 23S rRNA mutation:A2063G | Mycoplasma pneumoniae |
| N317 | Mycoplasma pneumoniae 23S rRNA mutation:A2063G | Mycoplasma pneumoniae |
| N321 | Mycoplasma pneumoniae 23S rRNA mutation:A2063G | Mycoplasma pneumoniae |
| N094 | NDM | Acinetobacter baumannii, Enterobacter cloacae |
| N166 | NDM | Pseudomonas aeruginosa, Klebsiella pneumoniae, Proteus mirabilis |
| N179 | NDM | Acinetobacter baumannii |
| N217 | NDM | Pseudomonas aeruginosa |
| N218 | NDM | Pseudomonas aeruginosa, Klebsiella pneumoniae |
| N031 | OXA-23 | Acinetobacter baumannii |
| N034 | OXA-23 | Acinetobacter baumannii |
| N044 | OXA-23 | Acinetobacter baumannii |
| N045 | OXA-23 | Acinetobacter baumannii |
| N047 | OXA-23 | Acinetobacter baumannii |
| N048 | OXA-23 | Acinetobacter baumannii |
| N050 | OXA-23 | Acinetobacter baumannii |
| N061 | OXA-23 | Acinetobacter baumannii |
| N064 | OXA-23 | Acinetobacter baumannii |
| N182 | OXA-23 | Acinetobacter baumannii |
| N183 | OXA-23 | Acinetobacter baumannii |
| N202 | OXA-23 | Acinetobacter baumannii |
| N299 | OXA-23 | Acinetobacter baumannii |
| N323 | OXA-23 | Acinetobacter baumannii |
| N031 | OXA-48 | Klebsiella pneumoniae |
| N031 | OXA-51 | Acinetobacter baumannii |
| N034 | OXA-51 | Acinetobacter baumannii |
| N044 | OXA-51 | Acinetobacter baumannii |
| N045 | OXA-51 | Acinetobacter baumannii |
| N047 | OXA-51 | Acinetobacter baumannii |
| N050 | OXA-51 | Acinetobacter baumannii |
| N061 | OXA-51 | Acinetobacter baumannii |
| N064 | OXA-51 | Acinetobacter baumannii |
| N078 | OXA-51 | Acinetobacter baumannii |
| N094 | OXA-51 | Acinetobacter baumannii |
| N179 | OXA-51 | Acinetobacter baumannii |
| N182 | OXA-51 | Acinetobacter baumannii |
| N202 | OXA-51 | Acinetobacter baumannii |
| N299 | OXA-51 | Acinetobacter baumannii |
| N313 | OXA-51 | Acinetobacter baumannii |
| N322 | OXA-51 | Acinetobacter baumannii |
| N323 | OXA-51 | Acinetobacter baumannii |
