# Supplementary figures and images for: Clinical diagnostic value of targeted next generation sequencing for lower respiratory tract infection: a retrospective study
Source: Front Cell Infect Microbiol. 2026 Jul 8;16:1713445. doi: 10.3389/fcimb.2026.1713445 (PMC13388218; doi:10.3389/fcimb.2026.1713445)

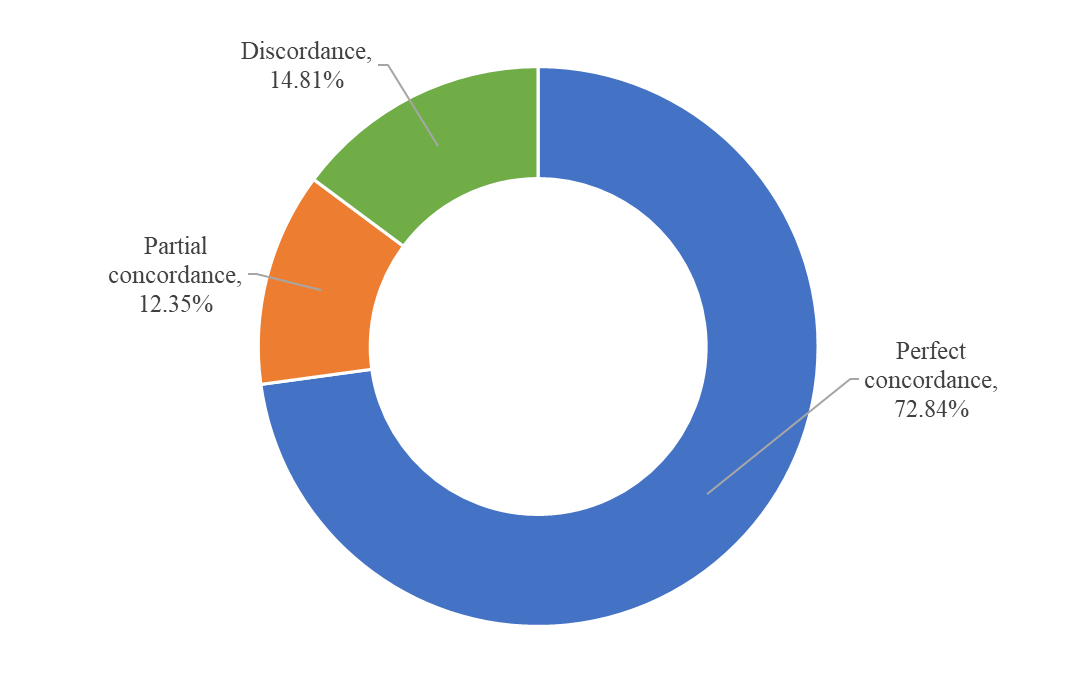


Supplementary Figure1. Comparison of the consistency between tNGS and CMTs.

Supplement: Supplementary file 2 [file SupplementaryFile1.docx]
